# Clinical Variation and Neuroimaging Patterns in Monozygotic Twins With Arrested X-Linked Adrenoleukodystrophy: A Case Report

**DOI:** 10.7759/cureus.86485

**Published:** 2025-06-21

**Authors:** Trevor A Leon, Steve M Nelson, Alex Gilman, Joseph Kluesner, Jon P Williams

**Affiliations:** 1 Department of Clinical Neurosciences, Boonshoft School of Medicine, Wright State University, Dayton, USA; 2 Department of Radiology, Wright-Patterson Air Force Base, Dayton, USA; 3 Department of Radiology, Boonshoft School of Medicine, Wright State University, Dayton, USA; 4 Department of Neurological Surgery, University of Arkansas for Medical Sciences, Little Rock, USA; 5 Department of Endocrinology, Wright-Patterson Air Force Base, Dayton, USA; 6 Department of Endocrinology, Boonshoft School of Medicine, Wright State University, Dayton, USA

**Keywords:** abdc1 gene mutation, clinical variation, imaging variation, monozygotic twins, x-linked adrenoleukodystrophy, x-linked adrenoleukodystrophy (x-ald)

## Abstract

X-linked adrenoleukodystrophy (ALD) is a peroxisomal disorder leading to neural and adrenal tissue deposition of very long-chain fatty acids. We report 23-year-old monozygotic twins who presented with neuropsychiatric concerns, fatigue, gait difficulty, lower extremity muscle stiffness, and urinary and bowel incontinence. One twin reported difficulty with concentration and mood and had more advanced difficulty with gait and incontinence as compared to his brother. Neurological examination identified weakness in proximal lower extremity muscles, hyperactive deep tendon reflexes in all extremities, positive Babinski sign, and spastic gait of differing severity between the patients. Magnetic resonance imaging (MRI) of the brain revealed T2 hyperintensity in the splenium of the corpus callosum in each subject. MRI of the spine revealed a normal signal in the cord; however, the thoracic segment was asymmetrically atrophied. No pathological enhancement was appreciated. Further testing revealed primary adrenal insufficiency and elevated hexacosanoic acid. Genetic testing confirmed a pathogenic variant in the *ABCD1* gene (c.796G>A (p. Gly266Arg), hemizygous). Neurological follow-up has revealed a persistent difference in symptom severity between the patients, which does not correlate with imaging findings or other biomarkers, despite the patients having the same mutation. Two previous reports of monozygotic twins with ALD were notable for progressive cerebral demyelination in only one patient of each pair. This report is unique in describing clinical and imaging variability in arrested cerebral ALD in these identical twins, underscoring the role of suspected non-genomic factors involved in the pathogenesis of this symptomatically diverse entity.

## Introduction

X-linked adrenoleukodystrophy (ALD) is a peroxisomal disorder that is characterized by the buildup and deposition of very long-chain fatty acids (VLCFAs) in the body, specifically neural and endocrine tissue such as the white matter of the brain, the spinal cord, and the adrenal cortex. X-linked ALD is caused by a mutation of the *ABCD1* gene found on the X chromosome. This gene codes for the adrenoleukodystrophy (ALDP) protein, which is a peroxisomal transmembrane protein responsible for transporting VLCFAs from the cytosol into the peroxisome for oxidation. Mutations in *ABCD1 *can cause dysfunction of this protein leading to intracellular accumulation of VLCFAs, which causes oxidative stress through the generation of reactive oxygen species, leads to mitochondrial dysfunction, triggers inflammation and immune activation, and disrupts lipid rafts and membrane-bound signaling pathways [1]. In males with ALD, there are three main presentations seen clinically: cerebral ALD (cALD), primary adrenocortical insufficiency, and adrenomyeloneuropathy (AMN). cALD manifests as a progressive cerebral inflammatory demyelination presenting with behavioral, cognitive, neurologic, and psychiatric symptoms that progress insidiously and usually result in severe disability or death. The onset of cALD typically occurs in early childhood but can arise into adolescence and adulthood. A second phenotype of ALD is the presentation of only primary adrenocortical insufficiency, otherwise known as Addison’s Disease. About 8% of males with ALD have Addison’s as the only clinical expression [2]. The third manifestation of ALD, AMN, presents with a slow-progressing paraparesis and pain in the lower limbs and common features of failure to control urinary sphincters and male impotence [1,3]. Loes et al. described five distinct MRI patterns of brain involvement in adrenoleukodystrophy. The most common pattern, seen in 66% of cases, involves the deep white matter of the parieto-occipital lobes and the splenium of the corpus callosum, often affecting the visual and auditory pathways. Less frequent patterns include frontal lobe or genu involvement (15.5%, mostly adolescents), frontopontine or corticospinal tract lesions (12%, mostly adults), cerebellar white matter changes (1%, mostly adolescents), and combined parieto-occipital and frontal white matter involvement (2.5%, mostly children) [4]. As a monogenic disorder with such variable clinical manifestations, familial and twin studies may provide additional insight into pathogenesis and/or clinical patterns in ALD. 

## Case presentation

We report two male, 23-year-old monozygotic (MZ) twins who presented for evaluation of recent and ongoing neurological and psychiatric concerns, including difficulties with gait, lower limb muscle stiffness, fatigue, as well as urinary and bowel incontinence. Both twins reported a multifaceted array of symptomatology and shared many similarities while maintaining unique independent phenotypic manifestations. Their family history is positive for a maternal great-grandmother who became wheelchair-bound in adulthood; multiple sclerosis was the initial clinical diagnosis. However, although no genetic testing was performed, ALD is suspected based on her clinical course. The patient’s maternal lineage includes German ancestry, while the paternal side is of Arab descent. The twins have an older brother who is asymptomatic and has tested negative for the *ABCD1* mutation.

Patient A presented with chronic symptoms that were rated more severely than patient B. A comparison of relevant neurological examination findings between the two patients is presented in Table 1. He reported having chronic constipation and difficulty with urination for as long as he can remember. He reported intense straining and administration of physical pressure to the abdomen to assist with urination and defecation. The patient, who was also a member of the cross-country team during high school, noted long-standing stiffness in his lower extremities. For the past three years, he has observed a decline in his motor strength and mental health, including a decrease in focus and attention. He reported consistent spasms and stiffness in his legs, with the right leg being worse than the left, and had right knee pain, specifically in the morning. In addition, he has noticed tingling and itching, mostly in his feet, and is no longer able to exercise due to fatigue that worsens throughout the day. This patient reported increasing symptoms of a depressive state, reporting little to no enjoyment in tasks that he once enjoyed, fatigue, and apathy. He also reported feeling distanced from close friends and family and finds trouble socializing and developing close emotional connections. Additionally, he mentioned episodes of negative self-talk and a significant episode of depression, during which he refused to eat. During this time, he contracted COVID-19 and interestingly experienced two to three days of newfound mental clarity. He is currently in school for computer engineering but describes an eye-twitching phenomenon while trying to read, making comprehension difficult. He also reported difficulty retaining auditory instruction.

**Table 1:** Selected neurological examination findings for twin patients. Persistent lower extremity examination performance, stable since initial clinic consult. Muscle weakness and gait discrepancies between the patients are indicated in bold font. Mental status, cranial nerve, upper extremity strength, as well as sensation and coordination testing were all within normal limits for the patients.

Exam component	Patient A	Patient B
Reflexes (bilaterally)		
Patellar	3/4	3/4
Achilles	Clonus	Clonus
Babinski	Present	Present
Motor strength (bilaterally)		
Hip flexion	4-/5	4/5
Hip extension	3/5	3/5
Hip abduction	3-/5	4-/5
Knee flexion	4/5	4/5
Knee extension	5/5	5/5
Ankle dorsiflexion	4+/5	5/5
Ankle plantarflexion	4+/5	5/5
Ankle eversion	4/5	5/5
Ankle inversion	5/5	5/5
Gait	B/L Trendelenburg; right foot inverted, pointed; decreased knee flexion; cannot stand on toes/heels; walks on toes	Right Trendelenburg; toe walking difficult

**Figure 1:**
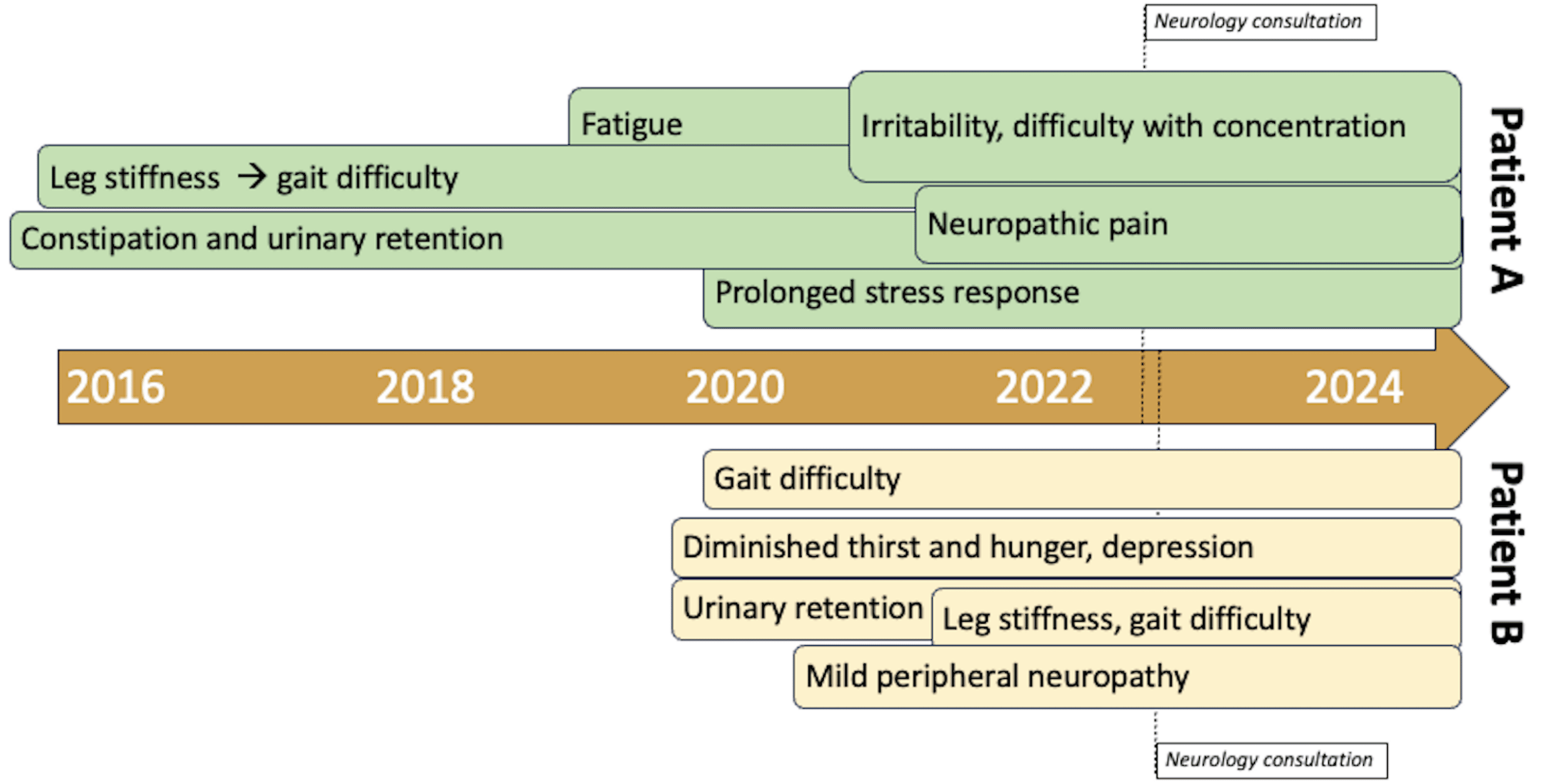
Symptomatic timeline. Clinical symptoms for patient A (above timeline, green boxes) and patient B (below timeline, tan boxes) are represented. Patient B reported minimal symptoms prior to 2020. The dotted line indicates the timing of the initial presentation to the neurology clinic.

The second twin (patient B) had a clinical burden consisting of difficulty walking, a prolonged stress response, difficulty urinating, minor peripheral neuropathy, and a diminished sense of hunger and thirst. The gait issues began 18-24 months prior to clinical presentation as the patient was finding himself struggling to keep up while walking with peers, despite being a competitive cross-country runner throughout middle and high school. He reported asymmetric motor involvement, specifically that his right leg is more affected than his left and notes persistent tightness in bilateral lower extremities with occasional tingling into the toes. In the mornings, the patient reported needing to warm up his muscles in both lower extremities prior to starting the day. He denied upper extremity symptoms. This twin also reported an abnormal stress response, noting that routine stressful situations can trigger a prolonged emotional response out of proportion to the situation, with difficulty moving on emotionally after the event. In addition, patient B reported feeling disconnected when interacting with others, little satisfaction with the completion of tasks and apathy. These worsened after graduating high school. Patient B reported worsening of physical manifestations coincide with episodes of depression. He noted that he must adhere to a schedule for eating and drinking due to having a diminished sense of hunger and thirst. He endorsed urinary retention, which is worst in the mornings, and reported that he applies mechanical pressure to the bladder to empty it. He denied any fecal incontinence or constipation. Figure 1 illustrates a symptom timeline highlighting the differences in the onset of clinical presentations between the two patients.

The twins individually underwent single gene sequence analyses, which subsequently showed a hemizygous c.796G>A (p.Gly266Arg) mutation of the *ABCD1* gene located on position q28 of the X chromosome. These analyses confirmed that the same mutation was present in both twins. Furthermore, lab tests were utilized to assess the presence of hexacosanoic acid, C26:0, a VLCFA, in each patient’s serum. Both were elevated at 2.24 for patient A and 2.63 for patient B (nl. 0-1.3 nmol/mL). Both the genetic mutation analyses and the serum VLCFA studies are consistent with a diagnosis of ALD. 

Both patients were referred to the endocrinology department to facilitate screening for adrenal insufficiency. A baseline morning cortisol was assessed on both patients and then cosyntropin was administered, followed by a reassessment of cortisol 60 minutes later. Cortisol did not significantly increase in either patient (7.60 mcg/dL for patient A and 3.24 mcg/dL for patient B), and remained below 15 mcg/dL (via a monoclonal immunoassay), making adrenal insufficiency likely. ACTH was then assessed in both patients and found to be significantly elevated, thus confirming a diagnosis of primary adrenal insufficiency. Patient A was started on hydrocortisone 10 mg in the morning and 5 mg in the afternoon; he noted a marked improvement in fatigue and some psychosocial symptoms after starting treatment. Patient B was also started on hydrocortisone, and most recently, we titrated the dose to 10 mg in the morning, 5 mg in the afternoon, and 5 mg in the early evening. He also saw marked improvement in fatigue symptoms after starting treatment.

Contrary to the majority of primary adrenal insufficiency cases, both patients were found not to have mineralocorticoid deficiency. Both patients denied salt craving or history of hypotension, had no history of hyperkalemia, and had normal renin values (typically elevated in states of mineralocorticoid deficiency). In ALD, VLCFAs accumulate in the adrenal cortex but are known to spare the zona glomerulosa in 50% of cases [5]. Table 2 presents the laboratory results.

**Table 2:** Laboratory evaluation for patients A and B. Elevated adrenocorticotropic hormone (ACTH) levels are present in both patients. Following exogenous ACTH stimulation, baseline cortisol levels did not increase as would be expected in normal hormonal physiology. Renin activity was normal.

Laboratory parameter	Patient A	Patient B	Reference range
ACTH	1411	744	7.2–63.3 pg/mL
Cortisol			
A.M.	7.53	3.65	4.46–22.7 mcg/dL
Post-ACTH stimulation	7.60	3.24	≥15 mcg/dL
Renin activity	0.685	0.574	0.167–5.38 ng/mL/h
Hexacosanoic acid, C26:0	2.24	2.63	0–1.3 nmol/mL
Gene sequence analysis, *ABCD1*	c.796G>A	c.796G>A	No mutation

**Figure 2:**
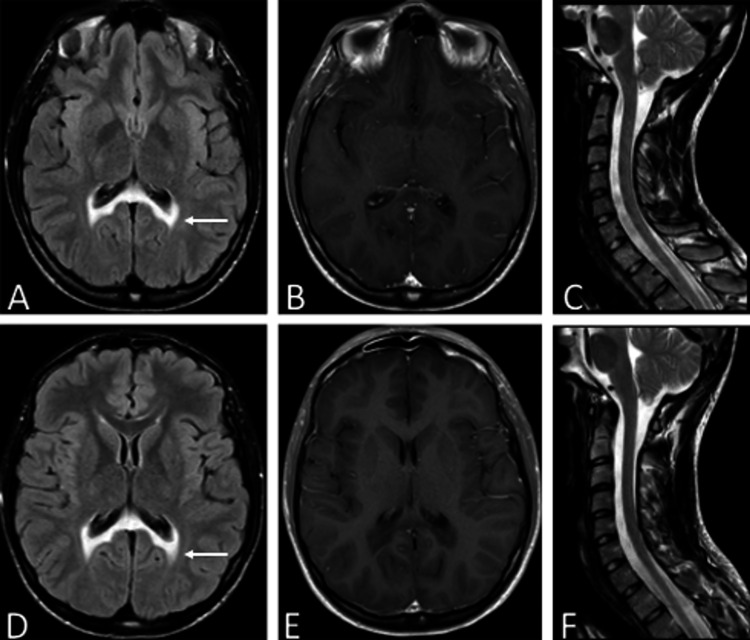
Imaging similarities in the brain and cervical spine of the twin patients. Brain MRI, axial T2 fluid-attenuated inversion recovery (A) and (D) and axial T1 post-gadolinium (B) and (E), demonstrating T2 hyperintensity in the splenium of the corpus callosum and the parieto-occipital deep white matter (white arrow: (A) and (D)) and no abnormal enhancement (B) and (E). Cervical spine MRI, sagittal T2 sequences demonstrating similar normal tissue signal and cord caliber (C) and (F). Patient A (A)-(C) and patient B (D)-(F).

**Figure 3:**
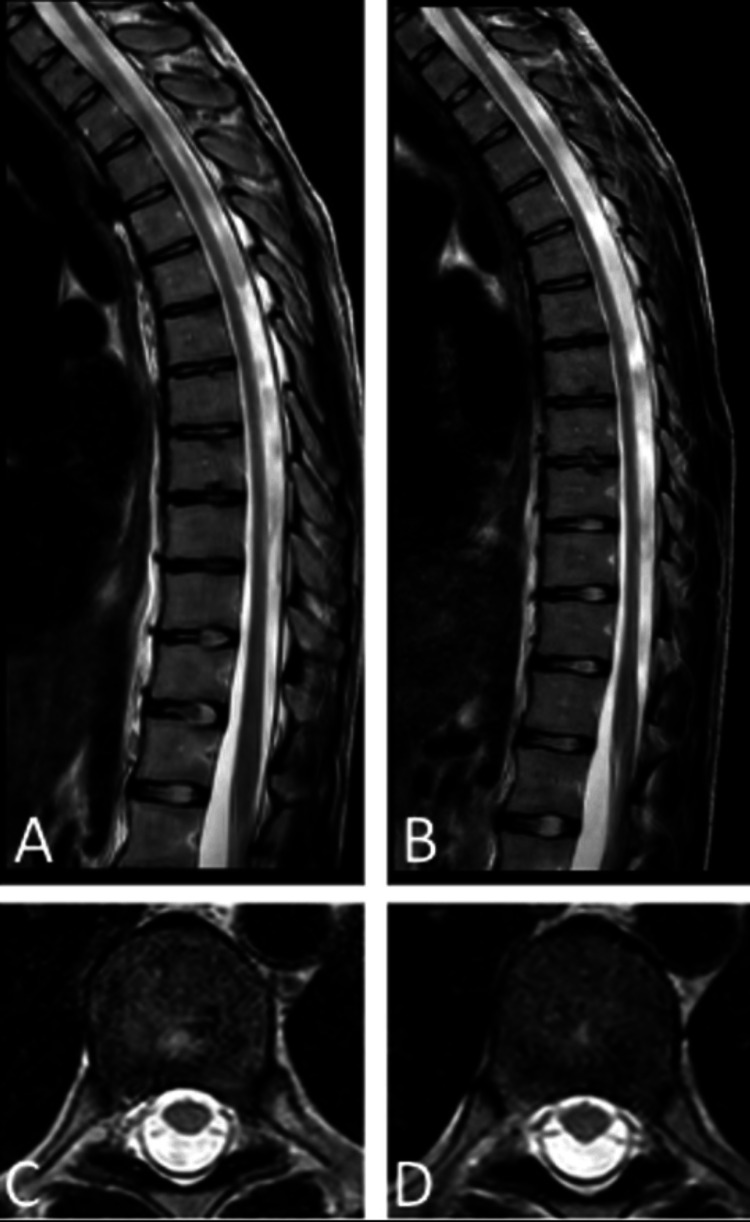
Thoracic spinal cord MRI of the twin patients. Thoracic spine MRI, sagittal T2 (A) and (B) consistent with atrophy. Axial sequences (C) and (D) at the level of the T9 vertebral body. Patient A (A) and (C) and patient B (B) and (D).

Imaging of the brain and spinal cord is shown in Figures 2, 3. For twins A and B, axial T2 fluid-attenuated inversion recovery (FLAIR) imaging reveals confluent disease involving the splenium of the corpus callosum as well as posterior periventricular white matter and involves only the posterior structures. There was no abnormal post-contrast enhancement for either twin on axial post-contrast T1 weighted imaging. The overall imaging features appear strikingly similar with respect to each twin (Figures 2A, 2B for twin A and Figures 2D, 2E for twin B). Additionally, the cervical spinal cord was normal in size and signal (Figure 2C for twin A and Figure 2F for twin B). 

Figure 3 shows an MRI of the thoracic spinal cord of the patients, both of which demonstrate an overall decrease in spinal cord caliber. When compared by radiology (S.N.) to an age-matched control, the spinal cord was notably atrophic at the level of T9 for patient A and T3-4 for patient B (age-matched control not shown).

The current treatment plan for the two patients involves careful clinical and radiographic monitoring, symptomatic management with medications (including baclofen, botulinum toxin, antidepressants, and hydrocortisone), and physical therapy. Should imaging detect the progression of cerebral injury, treatment options, such as hematopoietic stem cell transplantation (HCT) or gene therapy, would be considered. Ongoing monitoring and management is a coordinated multidisciplinary effort.

## Discussion

ALD is a monogenic disorder characterized by mutations in the *ABCD1* gene. Mutations in the *ABCD1* gene lead to dysfunction of the peroxisomal transport protein, ALDP, causing a failure of VLCFAs to cross into the peroxisome and undergo beta-oxidation. The accumulation of VCLFAs in the cytosol has been shown to lead to the death of microglia in the brain and the dysfunction of oligodendrocytes in the spinal cord. The dysfunction in these cells, in conjunction with oxidative stress and the release of pro-inflammatory cytokines, contributes to axonal degeneration in the central nervous system [6]. VLCFA accumulation also directly affects the adrenal glands leading to dysfunction of the adrenal cortex, particularly the zona reticularis and zona fasciculata [7]. 

The main clinical manifestations of ALD include a cerebral demyelinating form, an adrenomyeloneuropathic form, and a primary adrenocortical insufficiency. However, clinical presentation of the disease has been known to vary on an individual basis. The phenotypic variation in adrenoleukodystrophy (ALD) is well-documented. 

We reviewed the* ABCD1* Variant Registry, and there are 49 reports of the same 796G>A mutation harbored by the patients in this report [8]. These include patients from China, Germany, India, Japan, Norway, Portugal, Saudi Arabia, and the United States. All potential ALD phenotypes have been reported with this mutation. Some of these reports included family kindreds, but there is no clear reporting of the 796G>A mutation in monozygotic twins in the registry. In one Indian cohort, three of 17 unrelated patients had 796G>A and each had a different phenotype (childhood cerebral ALD-age 4.5, AMN-age 35, and adolescent cerebral ALD-age 11) [9]. A Japanese study reported that three of 40 had a phenotype of cerebral ALD and AMN, which were associated with 108S>L, 266Q>R, and 595W>X [10]. These reports clearly illustrate that the 796G>A mutation is not disproportionately associated with a particular phenotype, and the concurrent presentation of cerebral ALD and AMN can be associated with many different mutations within the *ABCD1* gene. 

A retrospective case series found that 64.7% of cases demonstrated clinical acceleration of pediatric cerebral ALD after COVID-19 infection [11]. The patients in the current study deny significant/known COVID-19 infection prior to presenting for neurology consultation, but symptom progression was temporally linked to the pandemic. 

Soardi et al. describe two young male patients affected by the cerebral demyelinating form of ALD. Both patients were positive for a mutation in p.Trp132Ter yet presented with differently. The first patient initially demonstrated adrenal insufficiency, which was then followed by moderate neurological symptoms. In the second patient, the neurological manifestations presented first and progressed rapidly without indications of adrenocortical insufficiency [12]. Another study reports two young male siblings presenting with the same mutation as one another (p.Gln316Pro). Both siblings showed neurological manifestations. However, one sibling manifested with neurologic symptoms that rapidly progressed, leading to his untimely death. This stark difference led the authors to conclude that genotype in ALD is not directly correlated with the disease phenotype [13]. The variation in ALD is believed to result from a combination of additional modifying genetic influences beyond ABCD1 mutation, epigenetic factors, and environmental contributors, which may include clinical or subclinical infectious, toxic, or unknown exposures [14]. Though ALD is a monogenic disorder, these additional factors may influence the severity and penetrance of the disease. 

These factors become especially pertinent in the case of monozygotic twins due to the presence of identical genotypes and mutations. For instance, a previous report of monozygotic twins with adult-onset ALD described one twin presenting with cerebral involvement and the other with adrenal insufficiency, though both exhibited myeloneuropathy [15]. Similarly, another report documented childhood (age 10) onset with MRI abnormalities and progressive neurological symptoms in only one of a pair of twins [16]. 

This report is unique in describing phenotypic differences in monozygotic twins with adolescent-onset ALD. Patient B experienced a delayed onset and less severe myeloneuropathic and neuropsychiatric symptoms compared to patient A. Despite these clinical differences, both patients exhibit strikingly similar cerebral imaging findings, which reveal arrested white matter injury. Notably, the patients also show regional variation in thoracic spinal cord diameter. The signal abnormality of the posterior white matter, including the splenium, likely accounts for the neurocognitive and behavioral problems. The variation in spinal cord thickness likely directly relates to spasticity and gait impairment, possibly with the lower thoracic abnormality in patient A accounting for the earlier onset of these symptoms.

## Conclusions

These cases highlight the complexity of ALD and the role that non-genetic factors play in phenotypic variability. Due to the near identical imaging findings, the study suggests that *ABCD1 *mutations in a syngeneic pair may have the most reproducible impact on the observable imaging changes in the central nervous system. However, despite identical mutations, the twins displayed significant differences in symptom severity underscoring the importance of comprehensive clinical assessment, as biomarkers alone cannot predict disease presentation or progression in ALD. Symptom expression and tempo may be modulated on an individual basis with the interplay between primary mutations, genetic modifiers, epigenetics, and environmental factors, which may involve clinical or subclinical exposures to infectious agents, toxins, or unidentified factors. These findings indicate the importance of individualized evaluation and treatment for patients with ALD regardless of mutation similarities. Future investigations of ALD are warranted, particularly in genomic and paraclinical correlates to symptom manifestation and clinical outcomes.
